# Automated, image-based disease measurement for phenotyping resistance to soybean frogeye leaf spot

**DOI:** 10.1186/s13007-022-00934-7

**Published:** 2022-08-16

**Authors:** Samuel C. McDonald, James Buck, Zenglu Li

**Affiliations:** 1 Institute of Plant Breeding, Genetics, and Genomics, and Department of Crop and Soil Sciences, University of Georgia, Athens, GA USA; 2 Department of Plant Pathology, University of Georgia, Griffin, GA USA

**Keywords:** Soybean, Frogeye leaf spot, Plant disease, Phytopathometry, Image analysis, ImageJ software

## Abstract

**Background:**

Frogeye leaf spot is a disease of soybean, and there are limited sources of crop genetic resistance. Accurate quantification of resistance is necessary for the discovery of novel resistance sources, which can be accelerated by using a low-cost and easy-to-use image analysis system to phenotype the disease. The objective herein was to develop an automated image analysis phenotyping pipeline to measure and count frogeye leaf spot lesions on soybean leaves with high precision and resolution while ensuring data integrity.

**Results:**

The image analysis program developed measures two traits: the percent of diseased leaf area and the number of lesions on a leaf. Percent of diseased leaf area is calculated by dividing the number of diseased pixels by the total number of leaf pixels, which are segmented through a series of color space transformations and pixel value thresholding. Lesion number is determined by counting the number of objects remaining in the image when the lesions are segmented. Automated measurement of the percent of diseased leaf area deviates from the manually measured value by less than 0.05% on average. Automatic lesion counting deviates by an average of 1.6 lesions from the manually counted value. The proposed method is highly correlated with a conventional method using a 1–5 ordinal scale based on a standard area diagram. Input image compression was optimal at a resolution of 1500 × 1000 pixels. At this resolution, the image analysis method proposed can process an image in less than 10 s and is highly concordant with uncompressed images.

**Conclusion:**

Image analysis provides improved resolution over conventional methods of frogeye leaf spot disease phenotyping. This method can improve the precision and resolution of phenotyping frogeye leaf spot, which can be used in genetic mapping to identify QTLs for crop genetic resistance and in breeding efforts for resistance to the disease.

**Supplementary Information:**

The online version contains supplementary material available at 10.1186/s13007-022-00934-7.

## Background

Frogeye leaf spot (FLS) is a foliar disease of soybean [*Glycine max* (L.) Merr] caused by the anamorphic fungus *Cercospora sojina* K. Hara that can hamper yield production in warm, humid climates by over 30% [1]. Since an outbreak of FLS in 1947 [2], it has remained an important soybean pathogen in the Southeast United States, and occasionally in parts of the Midwest. Though the disease can be controlled with fungicides or mitigated through good agronomic practices, such as crop rotation, crop genetic resistance has been the most successful method to prevent FLS infections [3].

Although FLS can appear on pods, stems, and seeds, it is primarily a foliar disease. Symptoms begin as water-soaked spots that progress to gray or brown lesions with reddish-brown margins [4]. Lesions are 1 to 5 mm in diameter but can merge to form large spots in severe infections [5]. In high moisture, leaf symptoms can appear within 48 h but typically are not observed for 8 to 12 days [4]. From 2012 to 2014, FLS was among the five most destructive soybean diseases in the southern United States [3], and in 2020 it was estimated to cause over 163 thousand tonnes of yield losses in the United States and 435 tonnes of loss in Ontario, Canada [6].

The importance of developing crop cultivars resistant to yield- and quality-reducing diseases has led to considerable interest in identifying quantitative trait loci (QTL) for resistance. The generation of high-quality phenotypic data is crucial to identifying QTL. Phenotype data that lacks accuracy or precision may lead to low precision QTL that span across large lengths of chromosomes with inflated QTL effects [7, 8]. Until recently, improving genetic studies has focused on increasing genotypic data and refining computational models. However, in recent years phenotypes have been the major constraint in identifying causative loci for important plant traits through QTL mapping and genome-wide association studies (GWAS) [9]. Most genetic studies for FLS resistance have relied on disease assays conducted in a greenhouse, which enables more uniform inoculation and the ability to conduct assays year-round, as opposed to the field. Several methods have been devised to measure the level of resistance of soybean genotypes to *C. sojina*. Mapping genetic resistance in ‘Davis,’ ‘Peking,’ PI 594774, PI 5944891, and other plant introductions (PI) have all relied on classifying plants or families as susceptible or resistant based on the presence or absence of lesions, respectively [10–13]. Quantitative methods of phenotyping have been developed as well, including ordinal scales and sets of standard area diagrams [14, 15], but have been used less frequently for genetic mapping research. Standard area diagrams offer increased precision over qualitative assessments, but accuracy may still be impacted by rater experience, the number of diagrams, and the quality of diagrams [16]. In the past, phenotyping severity of many plant leaf diseases have used 5-point ordinal scales, such as northern corn leaf blight [*Exserohilum turcicum* (Pass.) Leonard and Suggs] of maize (*Zea mays* L.) [17] and *Sclerotinia sclerotiorum* (Lib.) de Bary on common bean (*Phaseolus vulgaris* L.) [18]. Quantitative visual assessment of plant disease severity has been criticized for its reliability of accuracy and precision [19]. For example, allele effects for maize northern leaf blight resistance QTL were found to be dependent on raters, and QTL mapped with stepwise general linear models were inconsistent between raters [7]. Similarly, a comparison of visual assessment and image analysis for common bacterial blight (*Xanthomonas* spp.) of common bean suggested that the intervals in a 1 to 5 visual scale were unequal, which led to QTL effects being overestimated [20]. High-throughput and sensor-based phenotyping offer an opportunity for better estimates of QTL effects, more accurate assessments of heritability, and insights into certain genotype by environment (G × E) interactions, all of which have direct implications for genetic gain in a breeding program [21].

Obtaining phenotypic data has become the bottleneck in many plant breeding programs and genetic mapping studies. Thus, there have been extensive developments in phenotyping platforms that can increase accuracy, precision, resolution, or data collection speed. Accuracy and precision are two of the most important quality metrics by which phenotypic data are collected. Accuracy is typically defined as the closeness of a measurement to the true value, and precision is a measure of variability between measurements on the same sample [22]. On the other hand, resolution refers to the number of classes that a dataset has, with binary variables having the lowest resolution and continuous variables having a resolution that is only limited by the estimation or measurement method. Building high-resolution data collection techniques that retain accuracy and precision offer a deeper and more meaningful look into plant phenotypes. Especially in terms of plant diseases, the advent of sensor-based technology has allowed for the actual measurement of disease severity, in contrast to visual assessments that can only provide estimates [19, 20, 23, 24].

An additional advantage of image-based phenotyping is the immortalization of data, which can be referenced or reanalyzed as needed. This is especially important for phenotyping plant traits, as images can be reanalyzed if improved methods are developed after the experiment is terminated. Archiving images also allows the original data to be referenced.

Mapping crop genetic resistance QTL with sensing-based phenotyping systems allows for the collection of hundreds of samples with a digital camera, scanner, or other spectral sensor and subsequent automated analysis downstream [23]. Approaches from simple to complex have been devised to capture phenotypic data on disease severity. The simplest have utilized inexpensive digital cameras, color space transformations, and pixel value segmenting to identify, separate, and measure healthy tissues and diseased tissues. This technique of feature extraction has been used successfully to measure chlorotic and necrotic tissues on common bean infected with common bacterial blight [20] and to evaluate leaf rust diseases of cereals [24]. Building upon the basis of RGB image segmentation, hyperspectral imaging has been utilized to capture information outside of the range of visible lights, such as the use of normalized difference vegetation index (NDVI) for measurement of leaf rust in wheat (*Triticum aestivum* L.) [25]. Furthermore, computer vision made possible by deep learning has helped to derive meaningful information from images, such as a convolutional neural network that could detect 26 diseases of 14 crops with 99% accuracy [26]. Even though these more complex methods have been growing in popularity, systems using RGB images remain a practical choice because of their simplicity, low cost, and affordability.

The increased interest in image-based phenotyping has led to an increase in image analysis platforms. ImageJ [27] is a freely available and open-source java-based image processing software that is highly extensible. Plugins and macros can be built for the creation of custom tools and automatic processing of repetitive tasks. Other distributions of ImageJ, such as Fiji [28], bundle commonly used plugins with compatibility between ImageJ versions. The software is available for Windows, macOS, and Linux operating systems.

The objectives of this study were to develop an image processing pipeline that (1) produces reliable and repeatable measurements of soybean leaf area that is infected with frogeye leaf spot; (2) counts the number of frogeye leaf spot lesions present on a leaf; (3) can be automated to process hundreds of images, and (4) allows for data tracking from image acquisition to data output.

## Results

**Fig. 1:**
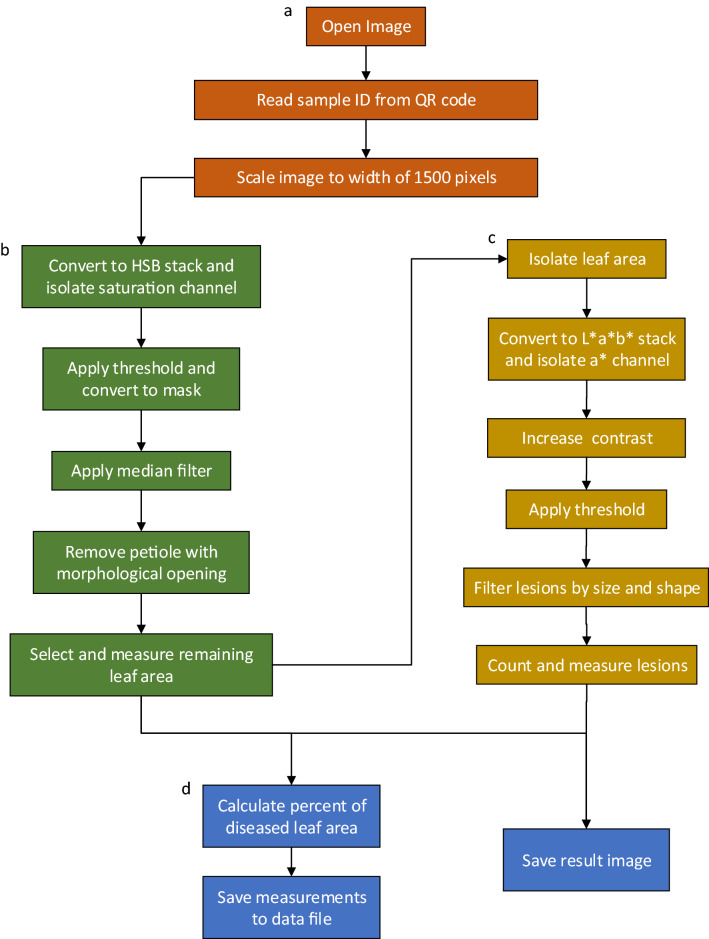
A method for measuring and counting frogeye leaf spot lesions on soybean leaves. The four main steps are **a** image preprocessing, **b** leaf segmentation, **c** lesion segmentation, and **d** calculation and data reporting

The image processing algorithm (Fig. 1) developed as an ImageJ macro can accurately count the number of frogeye leaf spot lesions on a soybean leaf and report the percent area of the leaf that is infected with lesions. The algorithm was developed using a set of 2096 images of soybean leaves with varying levels of frogeye leaf spot disease symptoms, ranging from no disease to highly infected. Before processing each image, the script reads a QR code that can be included in the image to track the data through the process and to the output. Without a QR code, it uses the name of the input image file to track and record data. The algorithm first isolates and measures the leaf area in pixels, then isolates, counts, and measures the area of lesions. When starting the program, the user has the option to run it in batch mode or single image mode. In batch mode, all image files in a directory will be processed according to set parameters. In single image mode, images are processed individually, and the user can adjust the segmentation and measurement parameters of the algorithm. The script saves a result image, in which the leaf and each individual lesion are outlined, and the decoded text from the QR code is printed on the image for data tracking. This image can be used to visually validate the results of the image processing system. The script saves a combined data file that contains the sample name, leaf area, lesion area, $$\frac{total lesion area}{total leaf area} \times 100$$, and lesion number for each sample. A summary file giving information on the images processed and the average time to process each image is also saved upon completion.

**Fig. 2:**
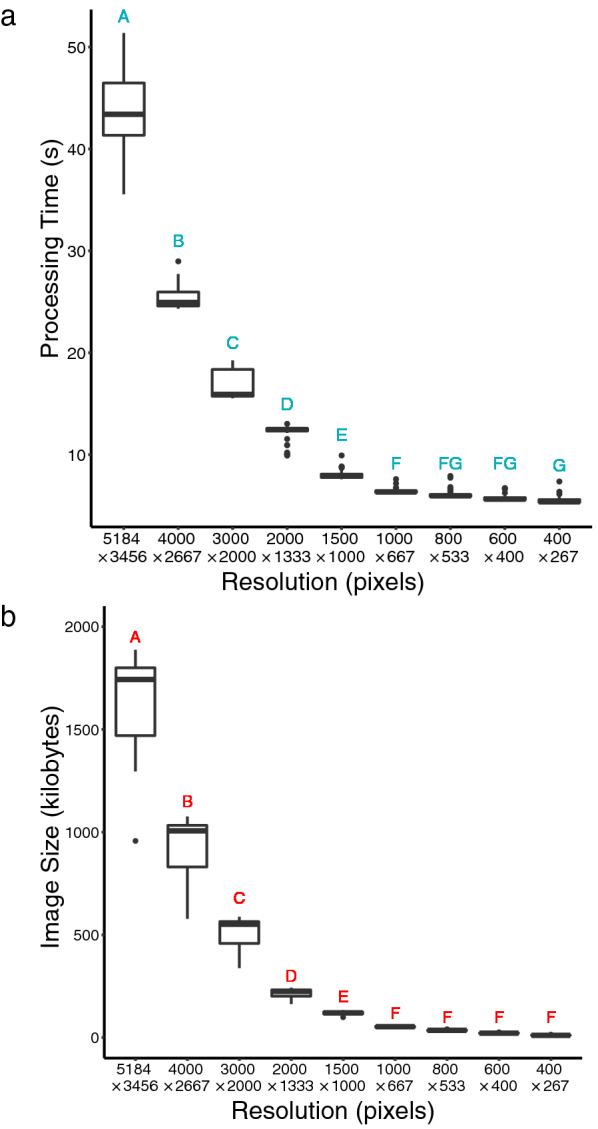
Boxplots and Tukey HSD showing the effect of image compression (image dimensions along X-axis) on **a** processing time and **b** result image size (n = 51)

In a test of different compression levels on 51 images, a resolution of 1500 × 1000 pixels was determined to be optimal, as there was a negligible effect on the precision of either trait. Pearson correlations for percent of diseased leaf area and lesion number were equal to 1.0 between uncompressed images (5184 × 3456 pixels) and images with a resolution of 1500 × 1000 pixels. Processing time was reduced from an average of 43.9 s for full resolution images to an average of 8.0 s for images compressed to 1500 × 1000 pixels (Fig. 2a). Similarly, average file size was reduced from 1661 to 117 kB when compressed (Fig. 2b). Lowering the image resolution further resulted in reduced correlation in measurements between compressed and uncompressed images (Table 1).

**Table 1:** Pearson correlations for percent diseased leaf area (upper) and lesion number (lower) traits between uncompressed images (5184 × 3456 pixels) and varying levels of compression (n = 51)

Image analysis trait	Compressed image pixel dimensions							
	4000 × 2667	3000 × 2000	2000 × 1333	1500 × 1000	1000 × 667	800 × 533	600 × 400	400 × 267
Percent diseased leaf area	1.00	1.00	1.00	1.00	0.99	0.99	0.99	0.98
Lesion number	1.00	1.00	1.00	1.00	0.99	0.99	0.99	0.99

**Fig. 3:**
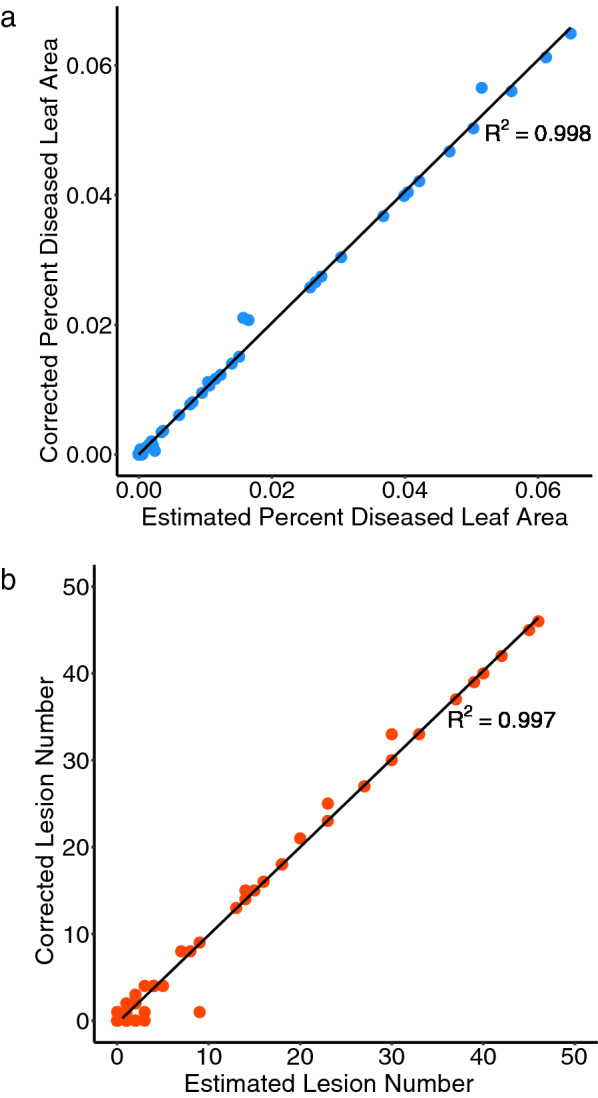
Pearson correlations between automatically measured values and manually corrected values for **a** percent of diseased leaf area and **b** lesion number traits (n = 75)

An analysis of 75 images representing the range of disease infection demonstrated that the automated image analysis method is highly concordant with manual measurements. Pearson correlations between automatic measurement and manual measurement were 0.998 and 0.997 for percent of diseased leaf area and lesion number, respectively (Fig. 3). When comparing the automated image analysis method and manual measurement, the percent of diseased leaf area deviated from the true value by 0.54% at most, with the average deviation being less than 0.05% from the true value. For lesion number, the maximum deviation was 8 lesions, and the mean deviation was 1.6 lesions from the true value. For both lesion area and lesion number, false positives were more prevalent than false negatives, meaning non-diseased tissues were occasionally classified as diseased. However, the average errors in lesion measurement and counting were small enough to be negligible. Six images showing a range of values for percent of diseased leaf area and lesion number estimated by automated image analysis and the manually corrected values, are included in Additional file 1.

To validate the image processing method for phenotyping FLS disease severity, the two traits generated from image analysis were compared to a visual assessment of disease severity on a 1–5 scale from 2096 plants phenotyped by a single rater. Spearman’s rank correlation coefficient was 0.85 between visual rating and percent of leaf area diseased and 0.83 between visual rating and lesion number. Percent of leaf area diseased and lesion number were highly correlated and had a Spearman’s rank correlation coefficient of 0.98.

## Discussion

A major challenge in improving frogeye leaf spot resistance in soybean is the generation of reliable phenotype data with sufficient resolution to quantify the variation of disease severity across genotypes. Analysis of RGB images for disease assessment has become a popular method for phenotyping plant leaf diseases. Although more advanced methods that use hyperspectral imaging or machine learning could also be highly accurate for phenotyping frogeye leaf spot disease severity, simpler methods using RGB cameras and image segmentation remain popular due to low startup costs for equipment and no need for expertise in hyperspectral sensing or machine learning. Image analysis offers accurate, precise, and repeatable measurements compared to visual methods that rely on standard area diagrams. Imaging the leaves of plants in a disease assay also allows for reanalysis if problems with the experiment are discovered downstream, or if improved methods of image analysis are developed in the future.

The fully automated image analysis tool enables precise measurements of leaf infection with detailed information on lesion number and size. On average, a set of 100 images can be analyzed in less than 15 min, minimizing the time between image acquisition and downstream data analysis. Additionally, the incorporation of a QR code reader enables data integrity in the result images and the output data files. In the proposed method, a result image is saved, which shows the leaf area that was measured and the area of each lesion that was counted and measured. The result image also prints a stamp of the decoded QR code if present and a timestamp at the end of the algorithm. This information allows for quick and easy confirmation of accuracy and ensures increased data integrity since values and measurements can be traced between the experiment and the input and output files.

Color space transformations have proven to be useful for feature segmentation in digital image analysis. By isolating one channel of a specific color space, each pixel of an image is reduced to a single dimension that represents the value at that pixel of that channel in the color space. In the leaf segmentation step, the saturation (S) channel of the HSB color space is used to segment the leaf area because the white background has a saturation near zero and the green leaf has a saturation typically higher than 100, so it is isolated in the 85 to 255-pixel value threshold. Similarly, transforming the RGB image into L*a*b* color space and isolating the a*-chrominance channel enables segmentation of the lesions away from the leaf. The ascending a*-chromatic value represents a shift from green to red, so reddish-brown lesions can be easily isolated from healthy, green leaf tissue in the image. Using fixed threshold values presents a risk due to variations between images that can be caused by lighting, leaf tissue color, or camera exposure. Even though these changes may be apparent in RGB color space, the L*a*b* color space is impervious to these variations, and the a*-chrominance channel values remain relatively constant across slight RGB value changes. The limitation with this method is that other reddish or brownish spots on the leaves, such as the damage caused by some insects or other diseases, cannot be distinguished from frogeye leaf spot lesions. Additionally, nutrient deficiencies may alter the color of the leaf, which may impact the ability to separate the tissue area diseased with FLS and healthy tissue. Many of the false positives observed in the tested image set could be attributed to non-FLS spots on the leaves that were counted as lesions. To mitigate this, a size selection step for lesions (i.e. lesions must be 16 more pixels in area) was implemented to prevent small flecks or other leaf spots from being measured. However, some false negatives were produced when the image processing did not detect very small FLS lesions. Thus, high-quality data still requires high-quality plant culture and greenhouse practices to minimize other causes of leaf discoloration.

To assess the accuracy of the image analysis algorithm, 75 images were processed and inspected for errors, which were corrected manually. For both lesion area and lesion number, there were more false positives than false negatives, on average. This means that the algorithm occasionally classified leaf areas that were not infected as being infected with FLS. As previously discussed, other causes of leaf discoloration, such as insect feeding or other leaf diseases, may lead to an overestimation of FLS disease severity. However, the average number of false-positive pixels was 10.90, which is below the lesion size threshold (16 pixels). This means that even though some extra leaf area may be classified as diseased, the average increase is less than the smallest allowable lesion size. Spearman’s rank correlations were also high between image analysis traits and the visual assessment trait, indicating that the image-based phenotyping performs similarly to conventional methods. It is important to note that a disease severity score given by a human rater may not be the true value. Thus, lower correlations between automatic measurement and visual disease scales may be due to deficiencies, biases, or lack of resolution in the visual assessment method and should not be the sole criteria for judging the accuracy of novel phenotyping platforms.

Automatic image segmentation and feature measurement offers a hands-off approach to processing images. Semi-automatic methods, in which the user can manually adjust parameters for each image offer more robust results but sacrifice time and ease. Users’ ability to fine-tune segmentation in semi-automatic image analysis methods may also introduce some bias, as many times, there is no clear-cut distinction between “diseased” and “healthy” pixels. Automatic segmentation also increases efficiency, as scripts can be built for hands-off analysis. Given these benefits and constraints, automatic image segmentation is preferable for phenotyping a large number of samples in FLS disease assays.

Image-based phenotyping platforms offer solutions to many of the challenges faced in plant phenotyping; they often are repeatable, fast, accurate, and have higher resolution than can be detected by a human rater. The methods presented here may be adapted and optimized in the future for other phenotyping applications. Developing accurate phenotyping methods with sufficient throughputs is a challenge in every crop system. If the symptoms of diseases, environmental stresses, or insect damage can all be easily detected in RGB images, phenotyping may be improved through a similar method of automatic segmentation and measurement.

## Conclusions

Identifying new sources of crop genetic resistance to FLS in soybean and incorporating genetic resistance into elite cultivars requires an improved method that produces accurate and detailed phenotype information. The proposed method of image-based FLS phenotyping is based on color space transformations and pixel value thresholding to segment key features from the image—namely the leaf and, if present, FLS lesions. Estimates of disease severity are acquired by measuring the percentage of leaf area that is diseased and counting the number of lesions on a leaf. The program can analyze an image in under 10 s with minimal computational requirements, allowing for the analysis of hundreds of images from a disease assay in an hour. Accuracy is comparable to visual methods of plant disease phenotyping and resolution is greatly increased. The reduction of false positives will be the first major goal in future research. However, the error in the tested dataset is small enough to have few practical implications. Using image processing to collect data on FLS disease severity is a convenient and accurate strategy that eliminates human-induced errors associated with conventional methods.

## Methods

### Plant materials, culture, and disease inoculation

Images used to develop and validate the phenotyping pipeline were taken from two different experiments. In the first experiment, plant materials consisted of 329 diverse soybean accessions selected from the USDA Soybean Germplasm Collection, of which four replicates were grown. The second experiment consisted of 180 recombinant inbred lines (RILs) that were derived from a ‘Forrest’ × ‘Davis’ cross, which were grown in six replicates. ‘Forrest’ is a susceptible soybean cultivar, while ‘Davis’ is a resistant cultivar that carries the major resistance gene *Rcs3* [29].

All disease assays were conducted in the Plant Pathology greenhouse at the University of Georgia Griffin campus in Griffin, GA. Experiments were laid out in a randomized complete block design, with two replicates being planted at a time. Four seeds were planted in a 10 cm square plastic pot and 12 pots were arranged in a 15-cell tray, leaving the middle three positions empty to maximize light distribution. After emergence, pots were thinned to two plants each. The greenhouse was maintained at approximately 27 °C during the day and 21 °C at night with 13 h of supplemental light during the winter and spring and 3 h during the summer from metal halide lamps. Plants were grown to the V2–V3 growth stage and inoculated with isolate S23 (race 8) of *C. sojina* from the University of Georgia *C. sojina* culture collection [4]. To produce inoculum, colonies of *C. sojina* growing on V8 agar media were flooded with 0.04% Tween-20 and lightly scraped with a scalpel to dislodge conidia. The conidia-Tween solution was passed through two layers of cheesecloth to remove large pieces of mycelium. The conidia concentration was measured on a hemocytometer and adjusted to 9 × 10^4^ spores × mL^−1^. At the time of inoculation, plants were moved to plastic-covered inoculation chambers to maintain humidity near 100% that were placed under 95% shade cloth to regulate temperature. In each chamber, 150 mL of prepared inoculum was evenly sprayed onto the trifoliolates of the plants. Inoculation was repeated 24 h following the same procedure. Plants remained in the inoculation chamber for additional 24 h after the second inoculation and then moved back to the greenhouse bench. Disease symptoms appeared on susceptible plants 14–21 days after inoculation.

### Image acquisition

Fourteen to 21 days after inoculation, RGB images were acquired with a Canon EOS Rebel T4i/EOS 650D digital single-lens reflex (DSLR) camera with a 17.9 megapixel resolution. The camera was mounted overhead 0.75 m above the subject. Images were taken on a plain white background with two LED lights set at 45° angles on either side. White balance of the camera was adjusted according to the manufacturer’s instructions before images were captured for the experiments. The camera was set to auto mode with flash disabled to automatically adjust shutter speed, aperture, and ISO speed. For each plant, the most diseased leaf was removed from the plant and placed on the white background. To keep the leaf flat, one piece of 20 cm × 20 cm nonreflective glass was placed on top of the leaf during imaging (ArtToFinish New York, USA). Each image also included a QR code for each sample indicating the experiment, pot number, and genotype, as well as a ruler for scale. Images were saved in JPEG format for analysis. To compare the results of the image analysis, the disease severity of each leaf was also estimated visually by a single rater using a 1–5 scale, where 1 = disease free, 2 = small lesions without a differentiated light center, 3 = < 10% of leaf area covered with lesions, 4 = ≥ 10% to < 20% of leaf area covered with lesions, and 5 =  ≥ 20% of leaf area covered with lesions. The same 1–5 scale has been used to phenotype soybean breeding lines at the University of Georgia due to simplicity compared to a 0–100% continuous scale, and similar scales are used to phenotype other crop leaf diseases [17, 18]. Visual estimates were based on a set of standard area diagrams [14].

### Image analysis

**Fig. 4:**
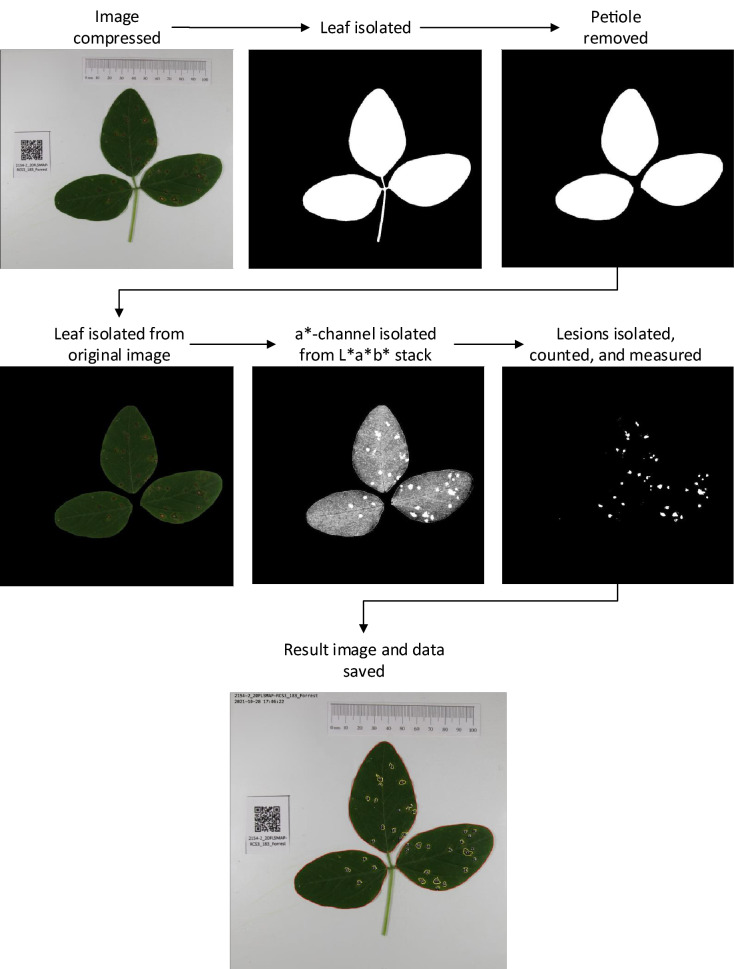
Image processing first isolates the leaf, removes the petiole, and measures the leaf. From the leaf, it then isolates, measures, and counts the lesions

The image analysis method (Fig. 4, Additional file 2) was developed in FIJI software [26], a free, open-source, and highly customizable distribution of ImageJ [27] for scientific image processing. To preprocess an image, it is first renamed and compressed. If a QR code was included in the image, it is decoded with the Barcode_Codec plugin [30], and the image file is renamed with the text decoded in the barcode. If no QR code is present, the image file name is used. Next, the image is rescaled so that the width is 1500 pixels. At this resolution, details of the leaves and lesions can be detected, but the storage space and computational power required are reduced.

After preprocessing, the RGB image is converted to a three-slice HSB (hue, saturation, brightness) color space stack to partition the leaf from the white background. In the saturation (S) slice of the HSB stack, segmentation with a threshold of 85 to 255 is applied, and the image is converted to a binary mask. A median filter with a radius of 4 is used to remove any remaining black pixels within the leaf area and white pixels in the background. To remove the petiole, morphological opening is used with an element of 9 to erode and subsequently dilate the binary image. By doing this, any structures in the image that are 18 pixels or narrower, such as the petiole, are removed and the leaf area remains almost unchanged. After the leaf has been isolated and the petiole has been removed, the remaining white pixels in the image are counted with the “Measure” function; this value is stored as the leaf area.

To isolate, measure, and count the lesions on each leaf, the compressed RGB is converted to a L*a*b* (lightness, a*-chrominance, b*-chrominance) color space stack, and the a*-chrominance channel is isolated. The background is first removed using the selection created in the leaf segmentation step, and the brightness and contrast of the leaf area are optimized to allow 0.35% of the pixels to become fully saturated. Next, a threshold of − 8 to 100 is applied to the a* channel to select the lesions. Then the “Analyze Particles” function is used to count the number of lesions with an area of 16 pixels or greater and a circularity of 0.3 or higher, where $circularity=4\pi \frac{area}{{perimeter}^{2}}$ and a value of 1 represents a perfect circle. The minimum area and circularity constraints minimize small debris or other irregularities less than 16 pixels or with a circularity value less than 0.3 from being classified as lesions. The “Measure” function is then applied to the selections to measure the pixel area of each lesion.

After processing each image, the percent of leaf area that is infected with lesions is calculated as $$\frac{total lesion area}{total leaf area}\times 100$$. Total leaf area, total lesion area, percent of diseased leaf area, and lesion number are saved in a combined file along with the sample name as determined by the QR code or input file name. For each image processed, a file containing the individual measurements of each lesion is also saved, as well as a result image that shows the leaf outline, the outline of each lesion, and the number of each lesion. This information can be used downstream to visually verify the accuracy of the image processing results.

### Optimization of image compression

To optimize the time to process each image and the amount of storage space required to store the result images, 51 images were tested using nine different resolutions in the image processing workflow. Compression levels ranged from 5184 × 3456 pixels (uncompressed original image) to 400 × 267 pixels, and steps in the image processing that rely on pixel dimensions were scaled accordingly. To determine the optimal compression, the lowest resolution that maintained an accurate count of lesions and measurement of lesion area was selected. Pearson’s correlations were used to compare the results from compressed images to the results of the uncompressed images for the percent of diseased leaf area and lesion number traits. One-way ANOVA and Tukey’s HSD were used to determine significant changes in image processing time and file size of the result image.

### Image-based phenotyping and comparison to visual phenotyping

To assess the agreement between image-based disease severity estimates and the actual disease severity, the 75 result images were visually inspected for healthy leaf areas detected as lesions or lesions that were not detected. To obtain an estimated true value for lesion area and lesion number in each image, lesions that were not detected and healthy areas that were marked as lesions were manually corrected and measured in ImageJ. Pearson correlations between the automatic measurements and true values were calculated for percent of diseased leaf area and lesion number in using the base R cor function. False positives were defined as any non-diseased area of the image that was marked as a lesion by the image processing software, and false negatives were defined as any lesion present in the image that was not marked by the software. False positives and false negatives were reported for lesion area and lesion number.

Spearman’s rank correlation coefficient was used to compare the image analysis traits with the 1–5 visual scale. Spearman’s rank correlation can be used to assess the relationship between continuous and ordinal variables and is based on the ranked value for each variable [31]. For 2096 samples that had data from the image processing and visual assessment traits, Spearman’s rank correlation coefficients were calculated for pairs of traits using the base R cor function.

## Supplementary Information

**Additional file 1.** Six result images with a range of disease severity. Automatically measured and manually corrected values for lesion number and lesion area are provided along with each image.

**Additional file 2.** The ImageJ plugin script designed to phenotype frogeye leaf spot of soybean by measuring the percentage of diseased leaf area and counting lesions.

## Data Availability

The ImageJ plugin is available for download at https://github.com/sam-mcdonald/FLS-Measure under GNU GPL Version 3 (https://doi.org/10.5281/zenodo.5719501). Requires ImageJ 1.45f or newer. The script is included as Additional file 2.
