# Supplementary material for: Automated, image-based disease measurement for phenotyping resistance to soybean frogeye leaf spot
Source: Plant Methods. 2022 Aug 16;18:103. doi: 10.1186/s13007-022-00934-7 (PMC9382788; doi:10.1186/s13007-022-00934-7)
Supplement: Supplementary file 1 — Additional file 1. Six result images with a range of disease severity. Automatically measured and manually corrected values for lesion number and lesion area are provided along with each image. [file 13007_2022_934_MOESM1_ESM.pdf]

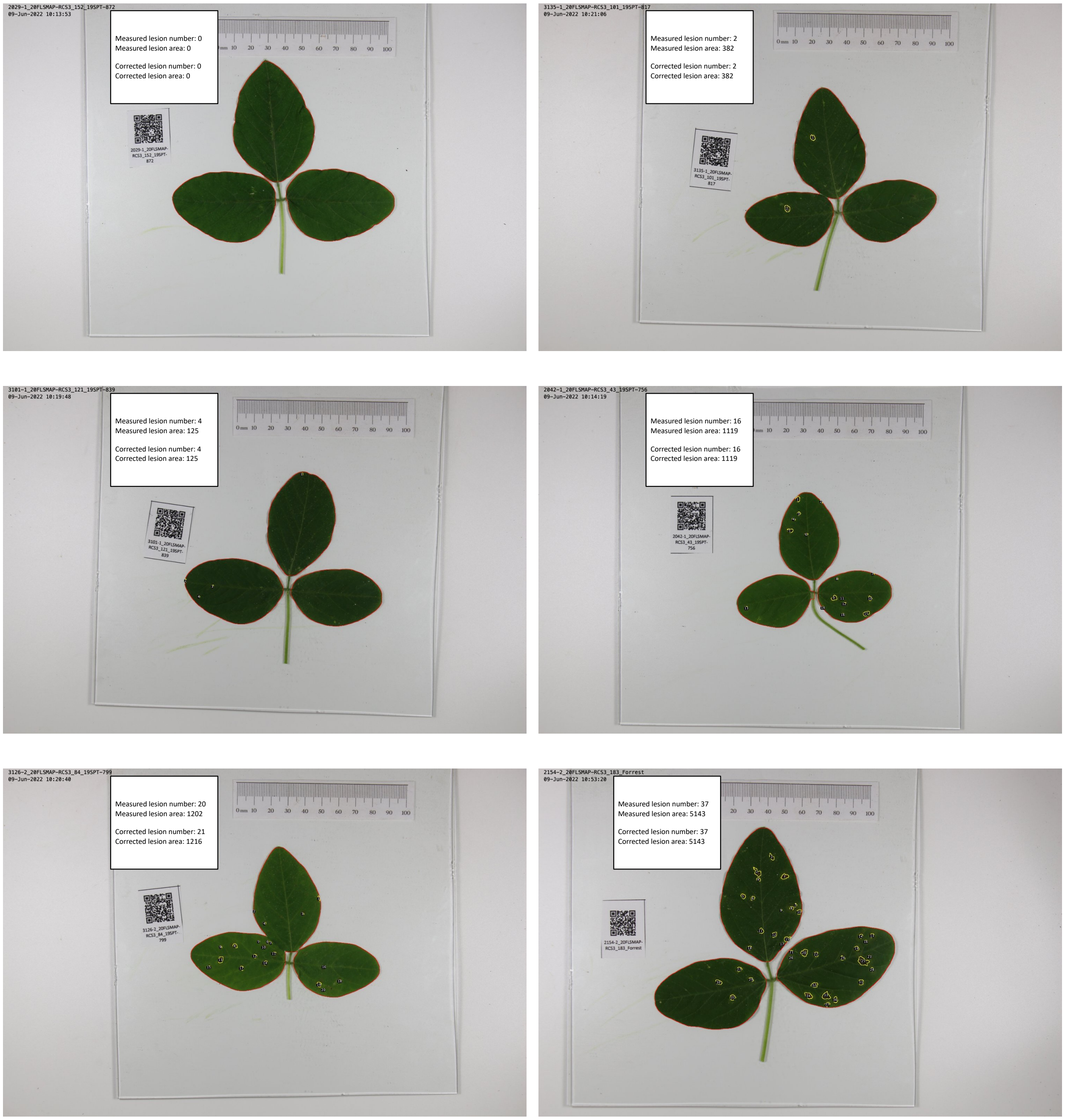

Additional File 1 Result images with disease severity ranging from completely resistant to highly susceptible to frogeye leaf spot. Lesion number and lesion area as measured automatically by the image analysis software, are indicated on each image. Corrected lesion number and lesion area were determined by making manual corrections to images after processing.
